# Weed-Associated Fungal Endophytes as Biocontrol Agents of *Fusarium oxysporum* f. sp. *cubense* TR4 in Cavendish Banana

**DOI:** 10.3390/jof7030224

**Published:** 2021-03-18

**Authors:** Dennice G. Catambacan, Christian Joseph R. Cumagun

**Affiliations:** 1 Institute of Weed Science, Entomology and Plant Pathology, College of Agriculture and Food Science, University of the Philippines, Los Baños 4031, Laguna, Philippines; dgcatambacan@up.edu.ph; 2 Research Information and Compliance Division, TADECO, Panabo City 8105, Davao del Norte, Philippines; 3 Molecular Phytopathology and Mycotoxin Research, University of Göttingen, Grisebachstrasse 6, 37077 Göttingen, Germany

**Keywords:** *Fusarium oxysporum* f. sp. *cubense* TR4, banana, weeds, fungal endophytes

## Abstract

The antagonistic activity of fungal endophytes isolated from weeds growing in Cavendish banana farms was determined against *Fusarium oxysporum* f. sp. *cubense* TR4 (*Foc* TR4) causing Fusarium wilt of Cavendish banana. Forty-nine out of the total 357 fungal endophytes from the roots of weeds exhibited antagonistic activity against *Foc* TR4. High inhibitory activity at 79.61–99.31% based on dual culture assay was recorded in endophytes *Lasiodiplodia theobromae* TDC029, *Trichoderma asperellum* TDC075, *Ceratobasidium* sp. TDC037, *Ceratobasidium* sp. TDC241, and *Ceratobasidium* sp. TDC474. All five endophytes were identified through DNA sequencing with 86–100% identity. Endophyte-treated Grand Naine and GCTCV 218 plantlets showed significantly lower disease incidence (*p* = 0.014), significantly lower degree of leaf yellowing (*p* = 0.037) and rhizome discoloration (*p* = 0.003). In addition, the cultivar Grand Naine was consistently highly susceptible compared with the tolerant cultivar GCTCV 218.

## 1. Introduction

Banana is among the world’s most valuable primary agricultural commodities and one of the most traded fruits worldwide. The Philippines which ranked the second largest exporter behind Ecuador, produced 3.4 million tons (USD 1.5 billion) in 2018 and contributed 90% of the total export volume in Asia [1].

Being grown in genetically uniform banana plantations, the crop is very susceptible to diseases, one of which is the most devastating disease Fusarium wilt caused by *Fusarium oxysporum* f. sp. *cubense* (*Foc*) Tropical race 4 (TR4). The disease is confirmed present in Cavendish banana plantations in Mindanao and has so far caused damage to many farms [2]. While there are already numerous studies to understand, and apply disease management strategies against Fusarium, there were few protocols which provided sustainable, long-term and commercial application in the field. Chemical methods have also not been found effective in the field [3]. As such, no sustainable control strategy exists for Fusarium wilt of banana.

The use of biological control agents as part of a sustainable and integrated plant disease management is increasingly becoming important especially in crops like Cavendish banana which are grown in monocultures with large pesticides inputs. Biological control of Fusarium wilt in banana has been studied for years [4]. Many studies revealed that suppressive sites have higher richness and diversity of microbial communities; with higher members of antagonistic microorganisms [5]. Endophytes are naturally occurring microorganisms integrally associated with their hosts. They have major roles in enhanced stress tolerance and protection against pathogens [6]. There have been observations in the field of healthy banana plants even if neighboring farms have been wilting due to *Foc* TR4 infection. It is plausible that this is influenced by beneficial microorganisms particularly those intimately associated with the bananas.

Weeds literally grow anywhere in diverse conditions. Like any other plants, weeds may have rich microbiota that may contribute to their survival in varying conditions. According to van Wees et al. [7], plant traits including disease resistance may be influenced by associated microorganisms which play a vital role in the ability of the weeds to thrive in suboptimal environments through contributing resistance and other functions related to plant-strengthening. There are more positive feedback interactions of weeds than crops with soil microorganisms leading to greater dependence on their associations [8]. They further reported that plant-associated microorganisms may be transmitted from weeds to other plants through different routes. This suggests that there is enormous value from these associated microorganisms of weeds in terms of resources of beneficial functions like antagonism against pathogens, plant growth promotion or interference of plant stress resistance.

This study aimed to identify fungal endophytes associated with roots of weeds growing in Cavendish banana farms, and determine their potential in controlling *Foc* TR4 in laboratory and greenhouse tests using the susceptible cultivar Grand Naine and the tolerant cultivar GCTCV 218.

## 2. Materials and Methods

### 2.1. Endophytic Fungi Source Collection and Isolation

Weeds growing in commercial and abandoned Cavendish banana farms were collected as sources of fungal endophytes. Root segments that appeared healthy or without necrosis [9] were cut into 1-cm segments. Following a modified method of Li et al. [10], roots were immersed in sterilized Type II laboratory water (ELGA Purelab 7000 Series, London, UK) for 10 s and sequentially immersed in 75% ethanol (1 min), 5% sodium hypochlorite (5 min), 75% ethanol (30 s), and four changes of sterilized Type II laboratory water for 1 min each. The surface-sterilized roots were placed equidistantly in streptomycin-amended potato dextrose agar or PDA (Himedia^®^, Mumbai, India). The parafilm-sealed plates were incubated at 26–27 °C with 12 h-dark/light cycle. The plates were monitored daily and mycelial tips of growing fungal colonies were transferred to PDA slants for pure culture. Each isolate was labeled properly with corresponding accession number.

### 2.2. Cavendish Banana Plantlets

Virus-indexed and tissue-cultured Cavendish banana cv. Grand Naine and GCTCV 218 were sourced from the Tissue Culture Laboratory—Research Information and Compliance Division of TADECO. Grand Naine is highly susceptible to Fusarium Wilt while GCTCV 218 is tolerant [11]. The rooted meriplants were planted in a 6” × 6” plastic bag with sterilized coco coir dust. Four weeks after, each plantlet was transplanted to an 8” × 12” plastic bag with heat-sterilized garden soil.

### 2.3. Foc TR4 from Cavendish Banana

A *Foc* TR4 isolate collected from an FW-infected Cavendish banana was used as control in laboratory and greenhouse tests. The infected banana manifested symptoms typical of Fusarium wilt disease, such as vascular discoloration of the pseudostem and intense yellowing of older leaves. This was confirmed as TR4 through DNA-based detection. Single spore culture of the isolate was generated and maintained as pure culture.

DNA from a *Foc* TR4 culture was isolated using a modified CTAB method [12]. The primer set FocTR4-F (5′-CACGTTTAAGGTGCCATGAGAG-3′) and FocTR4-R (5′-CGCACGCCAGGACTGCCTCGTGA-3′) by Dita et al. [13] specific for the detection of *Foc* TR4 was used in real-time PCR detection of the pathogen.

### 2.4. Identification of Fungal Endophytes

#### 2.4.1. DNA-Based Identification

Five fungal endophytes which gave the highest inhibitory activity against *Foc* TR4 based on dual culture assay were identified by molecular method. The fungal genomic DNA was extracted from the culture of the five fungal endophytes using the commercially available DNeasy Plant Minikit (Qiagen, Hilden, Germany) following the manufacturer’s procedures. Amplification of the internal transcribed spacer (ITS) region was performed with primers ITS1 (5′-TCC GTA GGT GAA CCT GCG G-3′) and ITS4 (5′-TCC TCC GCT TAT TGA TAT GC-3′) by White et al. [14] on a CFX96 Real-Time PCR machine (Biorad, CA, USA). In a 25 µL PCR reaction, 20 ng of genomic fungal DNA was mixed with 1× KAPA SYBR Fast qPCR master mix and added with primers at a final concentration of 0.4 µM. The following PCR profile was used: initial denaturation at 95 °C for 2 min, 35 cycles of denaturation at 95 °C for 1 min, annealing at 55 °C, extension at 72 °C for 1 min, and a final extension of 72 °C for 5 min. The PCR products were run on 1.25% agarose gel to check on amplified products. One sample per endophytic fungus was submitted for DNA sequencing. The unpurified amplicons were shipped to 1st Base Apical Scientific Sequencing Laboratory, Malaysia for DNA sequencing. TDC 474, one of the five fungal endophytes, was resubmitted to Macrogen, Korea due to poor chromatogram results. Chromatograms were manually edited and a consensus sequence was generated and subjected to Basic Local Alignment Search Tool (BLAST). Nucleotide sequences obtained were identified by comparison against the GenBank.

#### 2.4.2. Based on Morphological Characteristics

The endophytes were checked based on their morphological characteristics, such as colony growth on PDA and spores growing on hyphal tips. Microscopic structures were referred to the manual of Watanabe [15]. An agar block culture of each fungal endophyte was incubated for 4–5 days. The fungal endophytes growing on the slides and coverslips were stained with lactophenol cotton blue and examined in 100–400× light microscopy. Some endophytic fungi did not produce spores and hence were labeled as “non-spore-bearing fungi.” All endophytes were assigned to corresponding accession number.

### 2.5. In Vitro Assay on Antagonistic Activity of Fungal Endophytes

A preliminary test to check antifungal activity of 357 endophytic fungal isolates was done using 4-point assay involving inoculation of 5-mm discs of endophytes to four points in the plate with *Foc* TR4 disc at the center, was carried out. The plates were incubated at 26–27 ± 1 °C for 7 days and the antagonistic activity was observed as inhibition of the growth of the pathogen through niche or resource competition; or by antibiosis marked by a clear zone of inhibition. The fungal endophytes with observed antagonistic activities against TR4 were selected for further studies.

Out of the 357 fungal endophyte isolates, 49 isolates were evaluated further by dual culture method. The 5-mm mycelial discs from the actively growing edge of a 5-day old fungal endophyte and *Foc* TR4 culture were placed at 30 mm apart in PDA medium. As control, a mycelial disc of *Foc* TR4 alone was placed at 30 mm from the edge of the plate. Parafilm-sealed plates were incubated for 7 days at 26–27 ± 1 °C. The inhibition of radial growth of the pathogen was determined using the formula by Sonawane et al. [16]:

$$\mathrm{PIRG}\;\left(\%\right)=\;\frac{R1-R2}{R1}\times 100$$

where PIRG = percent inhibition of radial growth; R1 = growth radius of *Foc* TR4 in the control plate; R2 = growth radius of *Foc* TR4 towards the antagonist. Using the assessment by Soytong [17] as cited by Puig and Cumagun [18], the antagonistic activity of the fungal endophytes was described as >75% I—very high antagonistic activity; 61–75% I—high antagonistic activity; 51–60% I—moderate antagonistic activity; <50% I—low antagonistic activity.

There were three replications with five plates per replication.

### 2.6. Pathogenicity Test of Fungal Endophytes to Cavendish Banana

Five fungal endophytes which showed the highest antagonistic activity against *Foc* TR4 in in-vitro assay were inoculated to 5-week old banana plantlets to determine if they are pathogenic to Cavendish banana. The four non-spore-bearing endophytes were grown for 7 days in 10 mL potato dextrose agar to standardize the inoculum. Inoculation was done by burying cultures from half of a petri dish to four equidistant holes around the base of the plantlet. The spore-bearing endophyte was inoculated by drenching 50 mL spore suspension (10^6^ per mL) of a 7-day old culture to the base of the plantlets. Second inoculation was done a week after the first inoculation or to 6-week old banana plantlets. Eight weeks after, banana plantlets were assessed for the presence of leaf yellowing, rhizome discoloration and other symptoms of diseases.

### 2.7. Greenhouse Test of Fungal Endophytes vs. Foc TR4

Five fungal endophytes with highest antagonistic activity against *Foc* TR4 in dual culture assay were tested against the pathogen in pot test using cvs. Grand Naine and GCTCV 218. Four fungal endophytes were non-spore-forming hence cultured for 7 days in 10 mL PDA to standardize the inoculum. Inoculation of non-spore-bearing endophytes was done to 5-week old plantlets by burying cultures from half of a petri dish to four equidistant holes around the base of the plant. One fungal endophyte was spore-forming and inoculated to banana as drench at 50 mL per plantlet with 1 × 10^6^ spores per mL. Second endophyte application was done 1 week after the first endophyte application or to 6-week old banana plantlets. Two weeks after endophyte inoculation, *Foc* TR4 was inoculated to 8-week old banana plantlets by drenching 50 mL *Foc* TR4 solution adjusted to 1 × 10^6^ spores per mL. The sample plants were watered regularly to maintain high moisture necessary for infection. There were three replications with 15 plantlets per replication. Eight weeks after *Foc* TR4 inoculation, disease incidence and the degree of disease severity were assessed.

Disease incidence was calculated as:

$$\mathrm{Disease}\;\mathrm{Incidence}\;\left(\%\right)=\frac{\mathrm{Number}\;\mathrm{of}\;\mathrm{FW}-\mathrm{infected}\;\mathrm{samples}}{\mathrm{Total}\;\mathrm{number}\;\mathrm{of}\;\mathrm{samples}}\;\times 100$$

**Figure 1:**
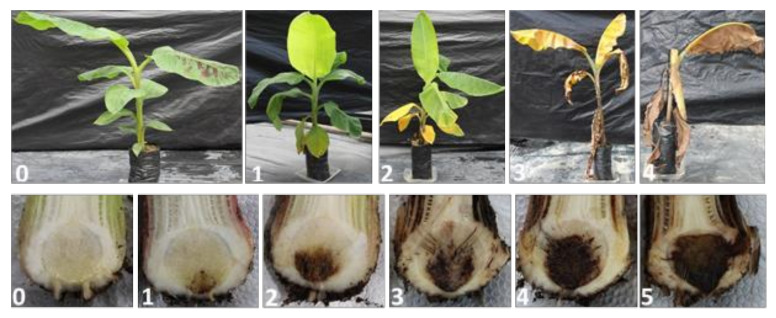
Rating scale for evaluation of external symptoms (upper panel) and internal rhizome discoloration (lower panel) caused by Fusarium Wilt to Cavendish banana in the greenhouse. Upper panel: 0—no yellowing; 1—initial yellowing mainly in lower leaves; 2—yellowing of all lower leaves with some discoloration of younger leaves; 3—all leaves with intense with intense yellowing; 4—plant dead. Lower panel: 0—corm completely clean; 1—isolated points of discoloration in vascular tissue; 2—discoloration up to 1/3 of the vascular tissue; 3—discoloration between 1/3 and 2/3 of the vascular tissue; 4—discoloration greater than 2/3 of the vascular tissue; 5—total discoloration of vascular tissue.

Disease severity based on leaf yellowing was assessed using a modified disease rating scale on leaf yellowing by Dita et al. [19] while the degree of rhizome discoloration was determined by longitudinally cutting the pseudostem to the rhizome and assessed using a modified rating scale by Carlier et al. [20] (Figure 1).

Percent disease severity was calculated as:

$$=\frac{\sum \;\left(\mathrm{Number}\;\mathrm{of}\;\mathrm{plants}\;\mathrm{in}\;a\;\mathrm{scale}\;\mathrm{category}\times \mathrm{specific}\;\mathrm{scale}\;\mathrm{category}\right)}{\mathrm{Total}\;\mathrm{number}\;\mathrm{of}\;\mathrm{samples}\times \mathrm{maximum}\;\mathrm{scale}\;\mathrm{category}}\;\times 100$$

### 2.8. Statistical Analysis

All data were statistically analyzed using analysis of variance (ANOVA) and Tukey’s HSD post-hoc test using IBM SPSS v.22 statistical software.

## 3. Results and Discussion

### 3.1. Fungal Endophytes from Weeds

Fifteen weed species belonging to 10 families were collected from commercial and abandoned Cavendish banana farms as sources of fungal endophytes. The three classes of weeds, grasses, broadleaves and sedges, were represented in the weed species collected from the sampling sites. The weed species were *Portulaca oleraceae* L., *Hedyotis corymbosa* (L.) Lam., *Cleome rutidosperma* DC., *Commelina diffusa* Burm. F., *Cyperus kyllingia* Endl., *Euphorbia hirta* L., *Eleusine indica* (L.) Gaertn., *Echinocloa colona* (L.) Link, *Brachiarya mutica* (Forsk.) Stapf., *Peperomia pellucida* (L.) H.B. and K., *Borreria*-like, *Ipomoea acquatica* Forsk., *Rottboellia cochinchinensis* (Lour.) W.D. Clayton, *Phyllantus amarus* Schum. and Thonn., *Vernonia cineria* L. Less.

**Figure 2:**
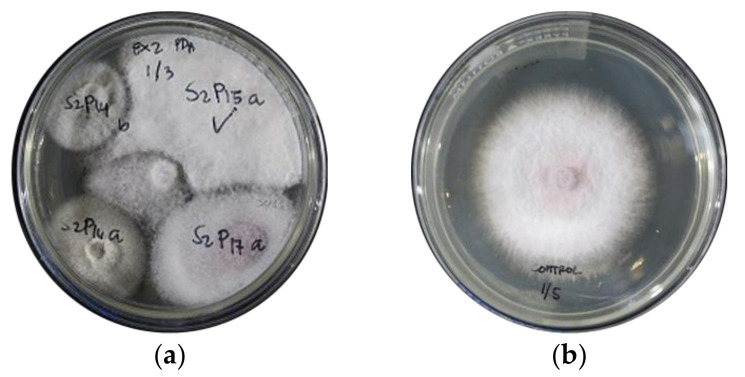
Four-point assay culture plate for the preliminary test of endophytic fungal isolates against *Fusarium oxysporum* f. sp. *Cubense* TR4 (*Foc* TR4). (**a**) Endophytic fungi at 4-point assay vs. *Foc* TR4, and (**b**) culture of *Foc* TR4 alone.

A total of 357 endophytic fungal isolates were recovered from 180 samples of weed species collected. Isolates which passed the preliminary test against *Foc* TR4 (Figure 2) were selected for identification based on colony growth and microscopic structures (Supplementary Figure S1). Among the 15 weed species, seven species harbored endophytic fungi. The broadleaf *Commelina diffusa* harbored the highest number of fungal endophytes with 18 fungal isolates. This was followed by both *Eleusine indica* and *Portulaca oleracea* with eight fungal isolates each, *Cyperus kyllingia* with six fungal isolates and *Brachiaria mutica* with five fungal endophytes. The least number of fungal isolates were recovered from *Echinocloa colona* and *Rottboellia cochinchinensis* with two fungal endophytes each. Almost 45% or 22 fungal endophytes were non-spore-forming while 55% or 27 isolates were spore-bearing. Identified fungal endophytes belonged to eight orders and nine genera in the fungal classification.

Moreover, 29% or 14 fungal isolates in this test were identified as *Fusarium*. All *Fusarium* isolates from weeds were confirmed as non-*Foc* TR4 when subjected to real-time PCR confirmation. Microscopic examination of the different *Fusarium* isolates indicates that they may be possibly belong to different species based on their morphological characters.

### 3.2. Antagonism of Fungal Endophytes Against Foc TR4

#### 3.2.1. In Vitro Assays

**Figure 3:**
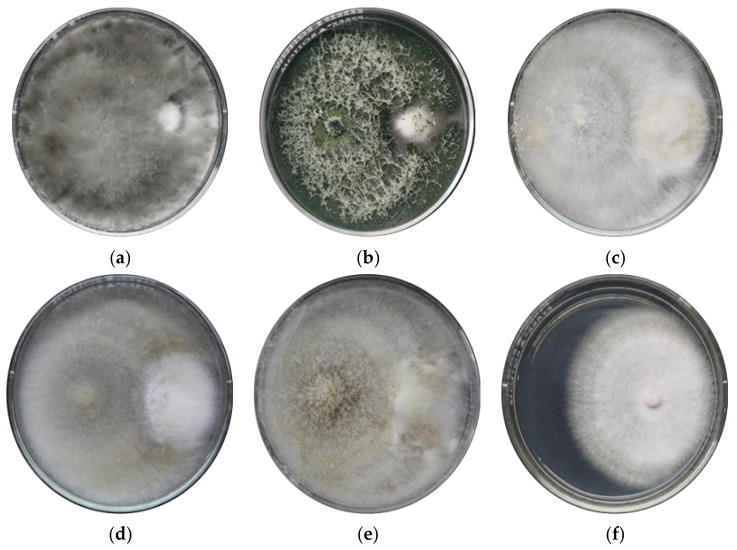
Dual culture plates of Foc TR4 and endophytic fungal isolates. (**a**) *Lasiodiplodia theobromae* TDC029, (**b**) *Trichoderma asperellum* TDC075, (**c**) *Ceratobasidium* sp. TDC037, (**d**) *Ceratobasidium* sp. TDC241, (**e**) *Ceratobasidium* sp. TDC 474, and (**f**) culture of *Foc* TR4 alone. Mycelial disc on the left side of the plate is the endophytic fungus while on the right is *Foc* TR4.

Preliminary results showed that out of the 357 endophytic fungal isolates recovered from weeds, 49 isolates exhibited inhibitory activity against *Foc* TR4. These were further tested against *Foc* TR4 using dual culture assay with results showing that all of the fungal endophytes exhibited varying degrees of antagonism against the pathogen. Based on Soytong [17] category on the antagonistic activity of fungal endophytes, 22 isolates exhibited moderate antagonistic activity with means ranging from 50.18% to 59.42% while another 21 isolates showed high antagonistic activity at 63.86–73.55% inhibition against *Foc* TR4. The remaining six isolates which included the top five fungal endophytes were categorized with very high antagonistic activity against the pathogen with means at 76.69–99.31%. The dual culture assay of five fungal endophytes which gave the highest inhibitory activity among the 49 fungal endophytes is shown in Figure 3. *Lasiodiplodia theobromae* TDC029 gave the significantly highest inhibition against *Foc* TR4 at 99.31%. *Trichoderma asperellum* TDC075 followed with 87.09% which was significantly comparable with the three *Ceratobasium* sp. Isolates TDC037, TDC241 and TDC474 at 80.63%, 79.69% and 79.61%, respectively. Results showed that weeds harbor fungal endophytes with antagonistic activity against *Foc* TR4. A total of 357 fungal endophytes were isolated from weeds. Forty-nine isolates were antagonistic towards *Foc* TR4. This may imply that with higher number of weed samples, an increased number and diversity of endophytes can be achieved.

#### 3.2.2. Pathogenicity of Fungal Endophytes to Cavendish Banana

To confirm if the five most effective fungal endophytes against *Foc* TR4 are non-pathogenic, they were inoculated to Cavendish banana cultivars Grand Naine and GCTCV 218. At 8 weeks after endophyte inoculation, rhizomes of inoculated banana plantlets were assessed for the presence of discoloration or any other symptoms of disease. All of the five fungal endophytes did not cause disease in both Grand Naine and GCTCV 218. Rhizome of the banana plantlets were all healthy with no visible necrosis and discoloration. Hence, this pathogenicity test confirmed that *Lasiodiplodia theobromae* TDC029, *Trichoderma asperelllum* TDC075, *Ceratobasidium* sp. TDC037, *Ceratobasidium* sp. TDC241, *Ceratobasidium* sp. TDC474 are not pathogenic to Cavendish banana cultivars Grand Naine and GCTCV 218.

#### 3.2.3. Screenhouse Test of Fungal Endophytes vs. *Foc* TR4

Eight weeks after *Foc* TR4 inoculation, there was a significant effect (*p* = 0.014) of the fungal endophytes application on the percent disease incidence of Fusarium wilt (Table 1). The control plant without fungal endophytes application gave a 100% disease incidence in both susceptible Grand Naine and tolerant GCTCV 218. On the other hand, fungal endophyte-treated banana plantlets gave lower Fusarium wilt incidence ranging from 85.39% to 91.18%. The fungal endophyte *Trichoderma asperellum* TDC075 gave the significantly lowest mean of 85.39%.

There was a significant difference (*p* = 0.000) between the two cultivars with the susceptible Grand Naine showing a higher mean of 97.39% disease incidence compared to GCTCV 218 at 83.69%. Data showed a high percent disease incidence even with the tolerant cultivar GCTCV 218 as it succumbed to the high *Foc* TR4 inoculum load. Even if endophyte-treated plantlets showed lower disease incidence, infection of Fusarium wilt can still be considered high. This confirms actual observations of the author in the field that tolerant cultivars, i.e., GCTCV 218 could succumb to Fusarium wilt in a highly infected soil due to high inoculum load. This is true in farms which do not practice eradication of Fusarium wilt-diseased plants in the field by leaving infected banana mats to rot in the field. This leads to the increase of *Foc* TR4 inoculum in the soil and causes further spread of the disease. A tolerant cultivar does not have a high chance of survival when confronted with a very high *Foc* TR4 inoculum. This highlights the importance of farm operations aimed at reducing inoculum load of *Foc* TR4 in the soil.

A significant effect of the fungal endophyte application (*p* = 0.037) on the degree of leaf yellowing was noted as shown in Table 2. Lower percent leaf yellowing was recorded from endophyte-treated Cavendish banana plantlets with means ranging from 26.72% to 30.64%. The fungal endophyte *Ceratobasidium* sp. TDC037 gave the significantly lowest mean on % leaf yellowing at 26.72%. The untreated control gave the highest mean of 36.77%. Percent leaf yellowing significantly differ (*p* = 0.000) between the two cultivars with a significantly higher mean of 36.44% in the susceptible Grand Naine.

**Figure 4:**
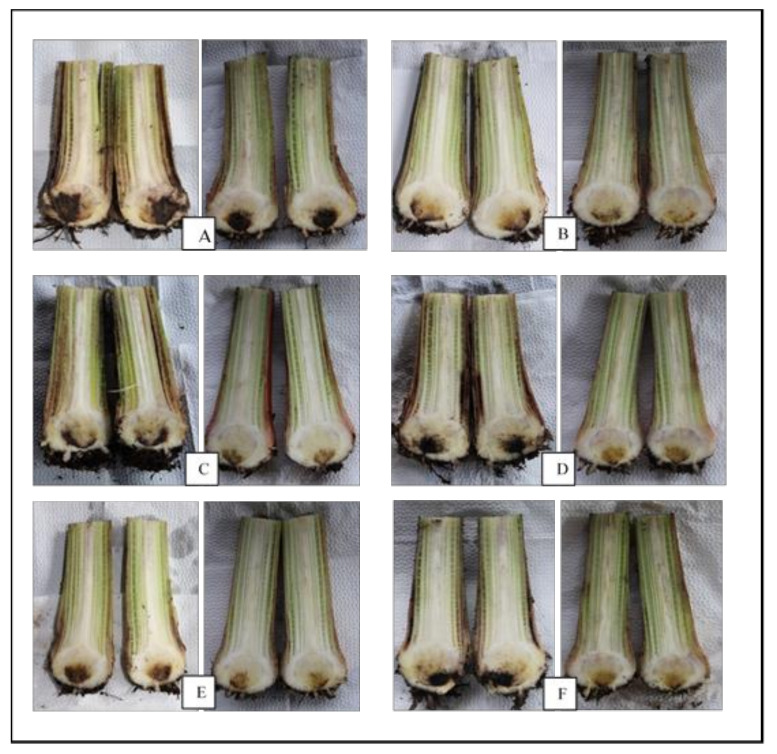
Effect of fungal endophytes from weeds on the degree of rhizome discoloration caused by Fusarium wilt on Grand Naine (**left**) and GCTCV 218 (**right**). (**A**) Control (untreated), (**B**) *Lasiodiplodia theobromae* TDC029, (**C**) *Trichoderma asperellum* TDC075, (**D**) *Ceratobasidium* sp. TDC037, (**E**) *Ceratobasidium* sp. TDC241, and (**F**) *Ceratobasidium* sp. TDC474.

Results on the degree of rhizome discoloration (Figure 4) showed that the untreated control gave the significantly highest mean (*p* = 0.003) of 44.71% which was statistically different from endophyte-treated Cavendish banana plantlets. Significantly lower comparable means ranging from 31.57% to 34.71% were recorded from banana plantlets treated with fungal endophytes (Table 3).

### 3.3. DNA-Based Identification of Fungal Endophytes

**Figure 5:**
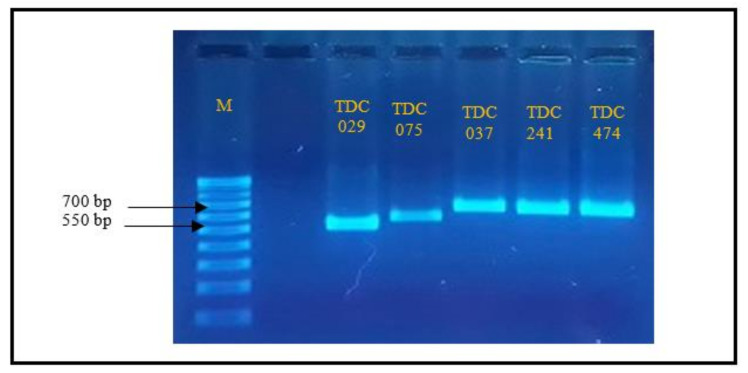
Amplified PCR product using ITS1 and ITS4 primers of the five endophytic fungi with high inhibitory activity against Foc TR4 in dual culture assay, Lanes: M-1k bp ladder, endophytic fungi codes indicated in lane.

Five fungal endophytes which showed high inhibitory activity against *Foc* TR4 based on in vitro assay were identified through DNA sequencing (Table 4). All samples amplified the internal transcribed spacer region (ITS region) in the ribosomal DNA. ITS region is the official DNA barcode for fungi [21]. Amplification of the five fungal endophytes yielded a single 550–700 bp DNA fragment (Figure 5).

Comparison of the DNA sequences with NCBI (National Center for Biotechnology Information) through BLAST showed that TDC029, TDC075, TDC037, and TDC241 gave 99.66–100% similarity to a known sequence. TDC474 on the other hand gave a lower percent similarity at 86%. All of the top isolates except TDC075 were non-spore-forming. Hence, DNA sequencing results provided accurate identification to endophytic fungal isolates which could not be identified morphologically due to the absence of identifiable structures such as fungal spores.

The three *Ceratobasidium* sp. isolates were recovered from separate weed species. *Ceratobasidium* sp. TDC037, *Ceratobasidium* sp. TDC241 and *Ceratobasidium* sp. 474 were isolated from *Rottboellia cochinchinensis, Commelina diffusa* and *Echinocloa colona*, respectively. On the other hand, *Lasiodiplodia theobromae* and *Trichoderma asperellum* were both isolated from the broadleaf weed *Commelina diffusa*. The endophytes *Lasiodiplodia theobromae* TDC029, *Trichoderma asperellum* TDC075, *Ceratobasidium* sp. TDC037, and *Ceratobasidium* sp. TDC241 were isolated from commercial Cavendish farm while *Ceratobasidium* sp. TDC474 was isolated from an abandoned Cavendish farm.

Monoculture production of Cavendish banana necessitates the large input of pesticides. This is necessary since growing the same crop all throughout long periods of time in vast lands means constant availability of hosts to pathogens. With the strict requirements for quality in the international market, banana growers rely on massive use of pesticides to maintain the level of diseases to a minimum such that quality of the fruits for export will be maintained. However, this raises environmental and safety concerns on the improper use of agricultural pesticides. Biological control against Fusarium wilt of banana which involves the use of antagonists as biological control agents offer promising potential as an effective strategy for the management of the disease. One of the most recent studies involving the use of biological control agents against Fusarium wilt of banana was of Castillo et al. [22] where the application of arbuscular mycorrhizal fungi (AMF) *Glomus* spp. protected Lakatan plantlets from *Foc* R4 and at the same time improved plant growth. The combined application of *Trichoderma harzianum* and *Glomus* sp. also delayed disease progression of Fusarium wilt in Lakatan.

Management of banana Fusarium wilt in which biological control plays an important part, the endophyte biology is an emerging field due to increasing research works on characterization of natural products, discoveries on their potential for biological control and crop production improvement [23]. Endophytes do not cause any immediate, overt or negative effects on the plant making them valuable and promising tools for agriculture [24]. According to Krings et al. [25], endophytic fungi have been associated with plants for over 400 million years. Each of the 300,000 species of higher plants is a host to many endophytes which are microorganisms that colonized living internal tissues of the host plant without causing adverse effects to the host [26]. Endophytes are reported as ubiquitous and have been studied in various hosts in diverse ecosystems such as in mosses [27], ferns [28], grasses [29], shrubs [30], deciduous and coniferous trees [31]. Weeds being abundant in nature can host a wide array of organisms including endophytes. For weeds, it is important to keep plant–microbiome interactions intact because weeds often thrive well in disturbed habitats like those in agricultural lands [32]. Weed-associated microorganisms may have roles in the weeds’ ability to thrive in suboptimal environments including plant resistance and other functions. Massessini et al. [8] reported that plant-associated microorganisms may be transmitted from weeds to other plants through different routes. The first author was able to isolate a variety of fungal microorganisms from weeds collected from a banana plantation in Mindanao, Philippines. A wide array of fungi was found thriving in the roots of weeds. The isolation of various fungi from the collected weeds raised the question on the role of these fungi as colonizers of roots. It was hypothesized that their presence in the roots of weeds or even in other plants served as competition against pathogenic organisms like *Foc* TR4. The question on whether these fungi can be introduced to economic crops like Cavendish banana and offer protection against destructive diseases is the basis of this research.

This study confirmed that fungal endophytes which are inhibitory against Fusarium wilt disease of Cavendish banana are present and can be isolated from weeds commonly growing in Cavendish banana farms. Furthermore, the higher number of recovered *Fusarium* endophytes from this test agrees with the organism’s wide distribution in many types of ecosystems. *Fusarium* spp. is a large genus of fungi commonly found in the soil and associated with plants and at the same time includes plant beneficial endophytes. Studies have been conducted on the potential of nonpathogenic *F. oxysporum* (np*Foxys*) to manage Fusarium wilt in banana. Fusarium wilt incidence in the greenhouse was significantly reduced in Cavendish banana plantlets treated with np*Foxys* isolates CAV 553, CAV 552 and CAV 563 isolated from Fusarium wilt- suppressive soils in South Africa [33]. Moreover, Thangavelu and Jayanti [34] reported the reduction of Fusarium wilt (*Foc* R1) by 80% in banana cultivar Rasthali when treated with two np*Foxys* Ro-3 and Ra-1. Despite the potential of nonpathogenic *Fusarium* being commonly associated with plants as endophytes, thorough evaluation should be done prior to wide-scale or commercial use of these endophyte as a biological control agent against diseases including Fusarium wilt as some strains can increase Fusarium wilt in banana [35] and the possibility of horizontal gene transfer that could transform np*Foxys* into pathogens [36].

All of the 49 endophytic fungi tested against *Foc* TR4 in the laboratory showed antagonistic activity against the pathogen through competition for space and nutrients as shown by the endophytes overgrowth vs. *Foc* TR4 in dual culture assay. Pathogens can be deprived of space and nutrients when beneficial organisms such as fungal endophytes colonize the shared habitat with the pathogen. Through competition of limited resources, growth of the pathogen is suppressed leading to reduction of the incidence and severity of the disease.

The top 1 endophytic fungus *Lasiodiplodia theobromae* based on its inhibitory activity against *Foc* TR4 in this test is an interesting microorganism. In a study by Pandi et al. [37], *L. theobromae* from the medicinal plant *Morinda citrifolia* was found to produce taxol, an effective antitumor agent. Taxol has been used for effective treatment of cancer such as refractory ovarian cancer, breast cancer, nonsmall cell lung cancer, AIDS-related Kaposi’s sarcoma, head and neck carcinoma, and other cancer types [38,39,40]. It was also isolated from the medicinal plant *Solanum nigrum.* Through metabolomics technology, the endophyte was found to produce different classes of bioactive metabolites such as sterols, y-lactone and plant auxin [41]. In a study by Eng et al. [42], submerged fermentation of *Lasiodiplodia theobromae* produced the hormone jasmonic acid. Jasmonates regulate plant immunity through induction of the expression of defensive genes against pathogen attack and feeding insects [43].

*Trichoderma* species have been used commercially for the management of many pathogens including *Fusarium* spp. In a study by Thangevalu and Gopi [44], greenhouse evaluation of combined application of a rhizospheric *Trichoderma* sp. NRCB + endophytic *Trichoderma asperellum* prr2 resulted in 100% reduction of Fusarium wilt disease and increased plant growth parameters up to 250%. Results of their study was further validated in the field through application at 15 days before planting, second and fourth month after planting, and resulted in a significant reduction of Fusarium wilt disease in the field as well as an increase in bunch weight. This means that the efficacy of biological control agents entails repeated and sustained application in the field.

Many isolates of *Ceratobasidium* sp. are originally isolated from roots of terrestrial orchid species. These fungi act as symbionts in orchids and play critical roles in germination of orchid seeds due to the orchid’s lack of sufficient food reserves [45]. *Ceratobasidium* spp. isolates have been reported to control *Rhizoctonia solani* in cotton [46], soybean [47], vegetable seedlings [48], and grasses [49]. *Ceratobasidium* spp. isolated from roots of Colombian orchids were tested for biocontrol of rice sheath blight caused by *Rhizoctonia solani.* The fungus *Ceratobasidium* spp. isolates significantly reduced disease expression of *R. solani* in rice seedlings when inoculated 3 days before *R. solani* [50].

Results of the test revealed the potential of fungal endophytes isolated from common weeds in Cavendish banana farms as biological control agents against the destructive disease Fusarium wilt. Promising research studies include optimization of the endophytes’ delivery to the plants in the field. To provide protection to Cavendish banana plantlets, endophytes may be applied to tissue-cultured plantlets while growing in the nursery through various methods; as cultures grown in corn grits, rice hull or as fungal suspension of spores and mycelia. Information on the effective amount or rates of endophytes against Fusarium wilt is necessary. It is important to check the effect of repeated or sustained endophytes application in the field towards the protection of growing Cavendish banana or towards reduction of Fusarium wilt incidences in the farm. Further studies to identify active metabolites in these endophytes as well as the optimization of its application to Cavendish banana plantlets in the field is in order. The fact remains that even with the diverse array of BCAs studied for Fusarium wilt control, tests on performance of these microorganisms in the field were not very common, hence, there is limited information on their long-term efficacy in the area. Most studies were short-term and mostly done in vitro. Due to this, Ploetz [51] recommended the use of tactics to foresee biological control failures in the field which would require better understanding of the interactions as well as realistic field evaluations.

## Figures and Tables

**Table 1:** Fusarium wilt incidence of Grand Naine and GCTCV 218 as affected by fungal endophytes from weeds.

Fungal Endophytes	Disease Incidence (%)			
	Grand Naine	GCTCV 218	Mean	STDEV
Control	100.00	100.00	100.00 ^b^	0.0
*Lasiodiplodia theobromae* TDC029	92.16	82.36	87.26 ^a^	12.0
*Trichoderma asperellum* TDC075	98.04	72.74	85.39 ^a^	15.9
*Ceratobasidum* sp. TDC037	98.04	84.31	91.18 ^ab^	12.1
*Ceratobasidum* sp. TDC241	98.04	84.32	91.18 ^ab^	8.9
*Ceratobasidum* sp. TDC474	98.04	78.43	88.23 ^a^	11.7
MEAN *	97.39 ^b^	83.69 ^a^		
STDEV	4.1	12.4		

cv (%)—8.95. * Mean difference between the two cultivars is significant at 0.01 level. Means having the same letter superscript are not significantly different at 0.05 level in Tukey’s HSD.

**Table 2:** Leaf yellowing of Grand Naine and GCTCV 218 caused by Fusarium wilt as influenced by weed-associated fungal endophytes.

Fungal Endophytes	Leaf Yellowing (%)			
	Grand Naine	GCTCV 218	Mean	STDEV
Control	44.12	29.41	36.77 ^b^	8.4
*Lasiodiplodia theobromae* TDC 029	36.27	21.57	28.92 ^ab^	9.8
*Trichoderma asperellum* TDC 075	38.24	23.04	30.64 ^ab^	9.9
*Ceratobasidum* sp. TDC 037	30.39	23.04	26.72 ^a^	5.3
*Ceratobasidum* sp. TDC 241	32.84	23.04	27.94 ^ab^	8.6
*Ceratobasidum* sp. TDC 474	36.77	19.12	27.94 ^ab^	10.1
MEAN *	36.44 ^b^	23.20 ^a^		
STDEV	6.4	5.2		

cv (%)—17.74. * Mean difference between the two cultivars is significant at 0.05 level. Means having the same letter superscript are not significantly different at 0.05 level in Tukey’s HSD.

**Table 3:** Rhizome discoloration of Grand Naine and GCTCV 218 caused by Fusarium wilt as influenced by weed-associated fungal endophytes.

Fungal Endophytes	Rhizome Discoloration (%)			
	Grand Naine	GCTCV 218	Mean	STDEV
Control	56.08	33.33	44.71 ^b^	13.8
*Lasiodiplodia theobromae* TDC029	41.96	21.18	31.57 ^a^	12.6
*Trichoderma asperellum* TDC075	47.06	22.36	34.71 ^a^	13.6
*Ceratobasidum* sp. TDC037	41.18	24.32	32.75 ^a^	10.0
*Ceratobasidum* sp. TDC241	41.18	22.35	31.77 ^a^	11.9
*Ceratobasidum* sp. TDC474	43.53	22.75	33.14 ^a^	12.0
MEAN *	45.17 ^b^	24.38 ^a^			
STDEV	7.9	5.13			

cv (%)—15.59. * Mean difference between the two cultivars is significant at 0.05 level. Means having the same letter superscript are not significantly different at 0.05 level in Tukey’s HSD.

**Table 4:** DNA-based identification of five weed-associated fungal endophytes with highest inhibitory activity against *Foc* TR4 in dual culture assay.

Code	Closest Match	NCBI Accession NO.	Identity (%)
TDC029	*Lasiodiplodia theobromae*	MK369927.1	100.00
TDC075	*Trichoderma asperellum*	MF061791.1	99.79
TDC037	*Ceratobasidium* sp.	KP089995.1	99.83
TDC241	*Ceratobasidium* sp.	KY965395.1	99.66
TDC474	*Ceratobasidium* sp.	KT265715.1	86.00

## Data Availability

Not applicable.

## Supplementary Materials

The following are available online at https://www.mdpi.com/2309-608X/7/3/224/s1, Figure S1: Colony growth on potato dextrose agar (PDA) and microscopic structures of fungal endophytes from roots of weeds growing in Cavendish banana farms.
