# Supplementary material for: Weed-Associated Fungal Endophytes as Biocontrol Agents of Fusarium oxysporum f. sp. cubense TR4 in Cavendish Banana
Source: J Fungi (Basel). 2021 Mar 18;7(3):224. doi: 10.3390/jof7030224 (PMC8003220; doi:10.3390/jof7030224)
Supplement: Supplementary file 1 [file jof-07-00224-s001.pdf]

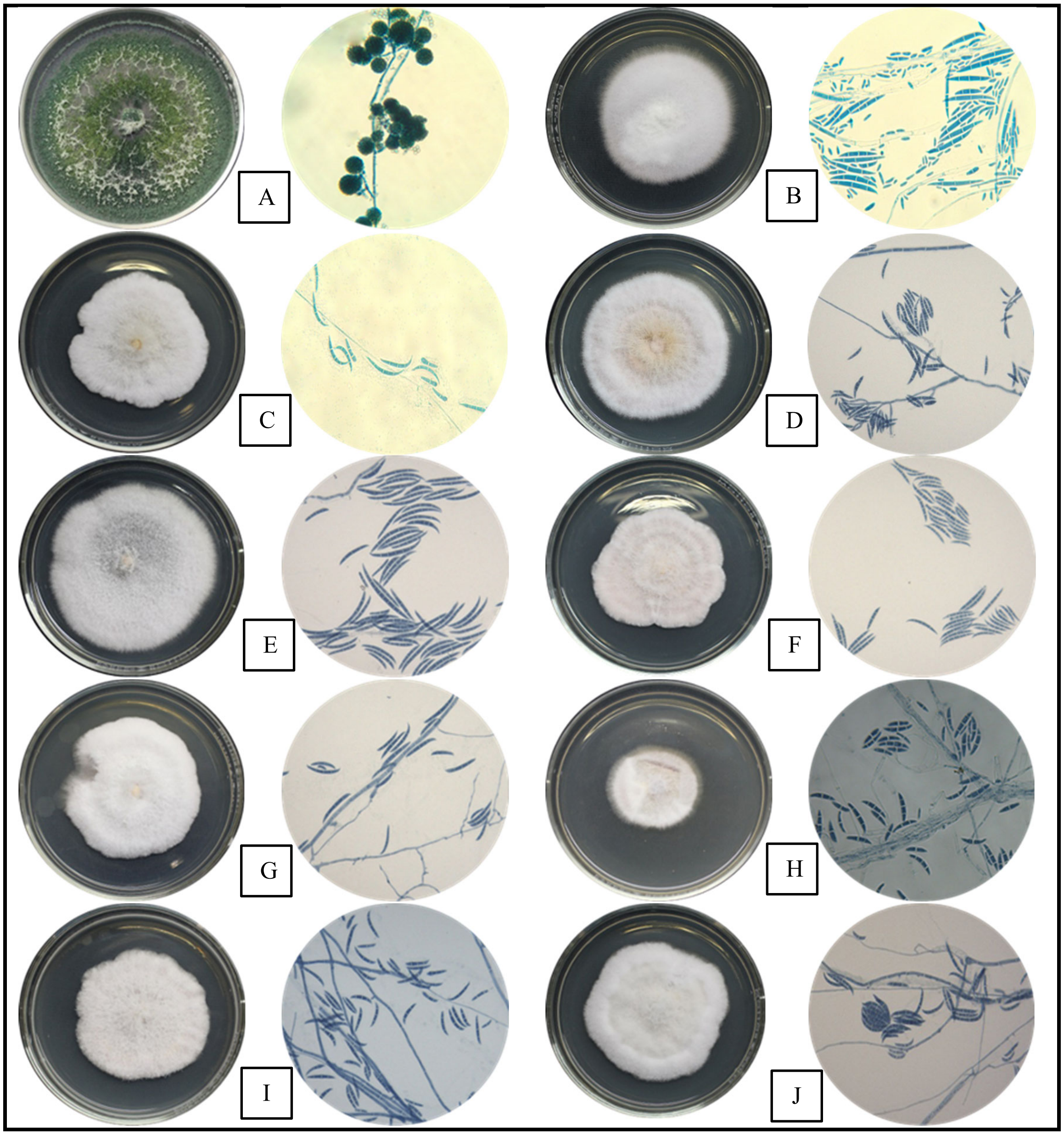

Supplementary Figure 1. Colony growth on potato dextrose agar (PDA) and microscopic structures of fungal endophytes from roots of weeds growing in Cavendish banana farms. A) *Trichoderma asperellum* TDC075, B) *Fusarium* TDC520, C) *Fusarium* TDC174, D) *Fusarium* TDC014, E) *Fusarium* TDC205, F) *Fusarium* TDC111, G) *Fusarium* TDC153, H) *Fusarium* TDC686, I) *Fusarium* TDC148, and J) *Fusarium* TDC182, (400x).

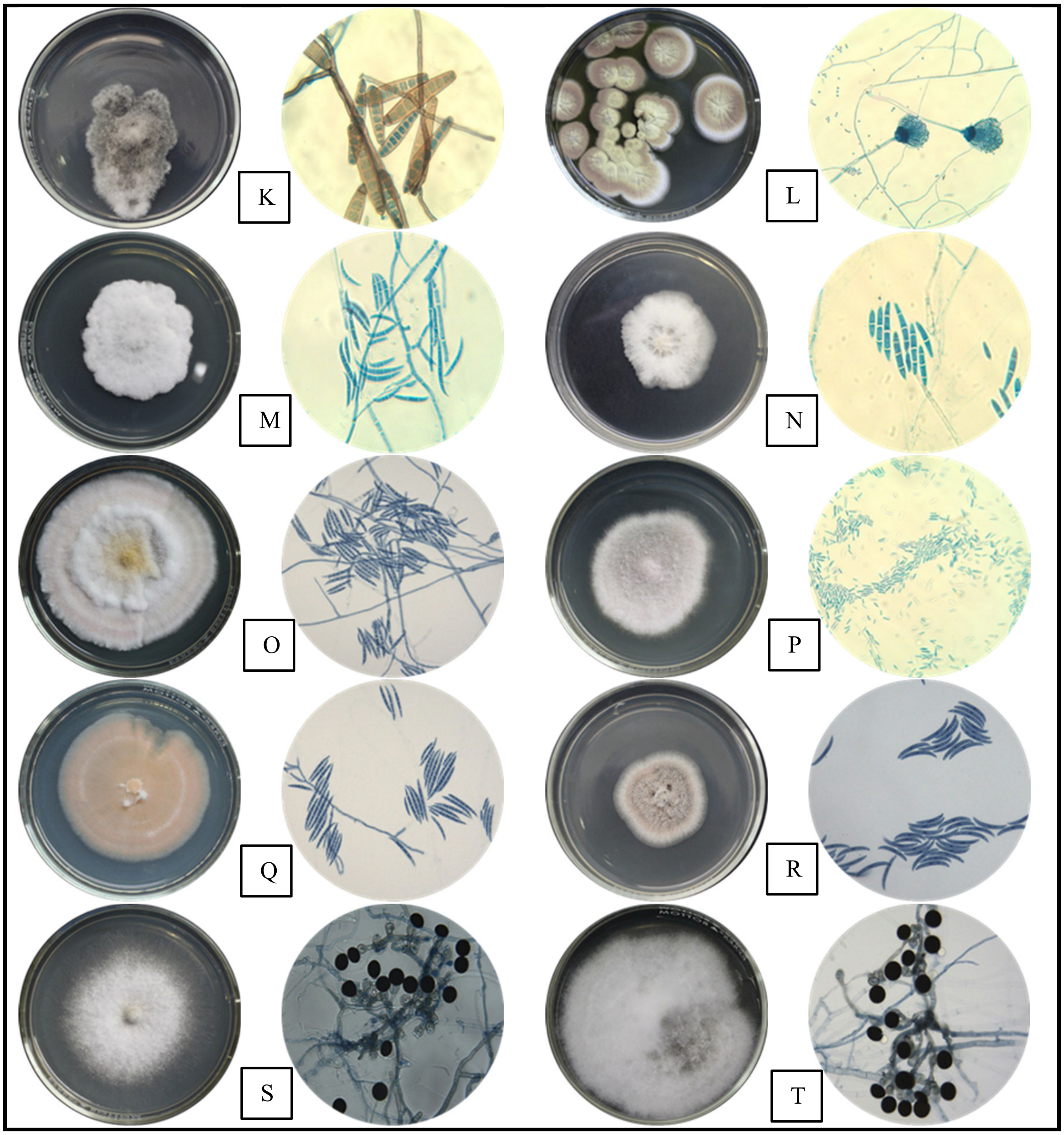

Supplementary Figure 1….continued.: K) *Helminthosporium* TDC158, L) *Penicillium* TDC099, M) *Fusarium* TDC134, N) *Fusarium* TDC682, O) *Fusarium* TDC100, P) *Colletotrichum* TDC260, Q) *Fusarium* TDC044, R) *Fusarium* TDC412, S) *Nigrospora* TDC107, and T) *Nigrospora* TDC225, (400x).

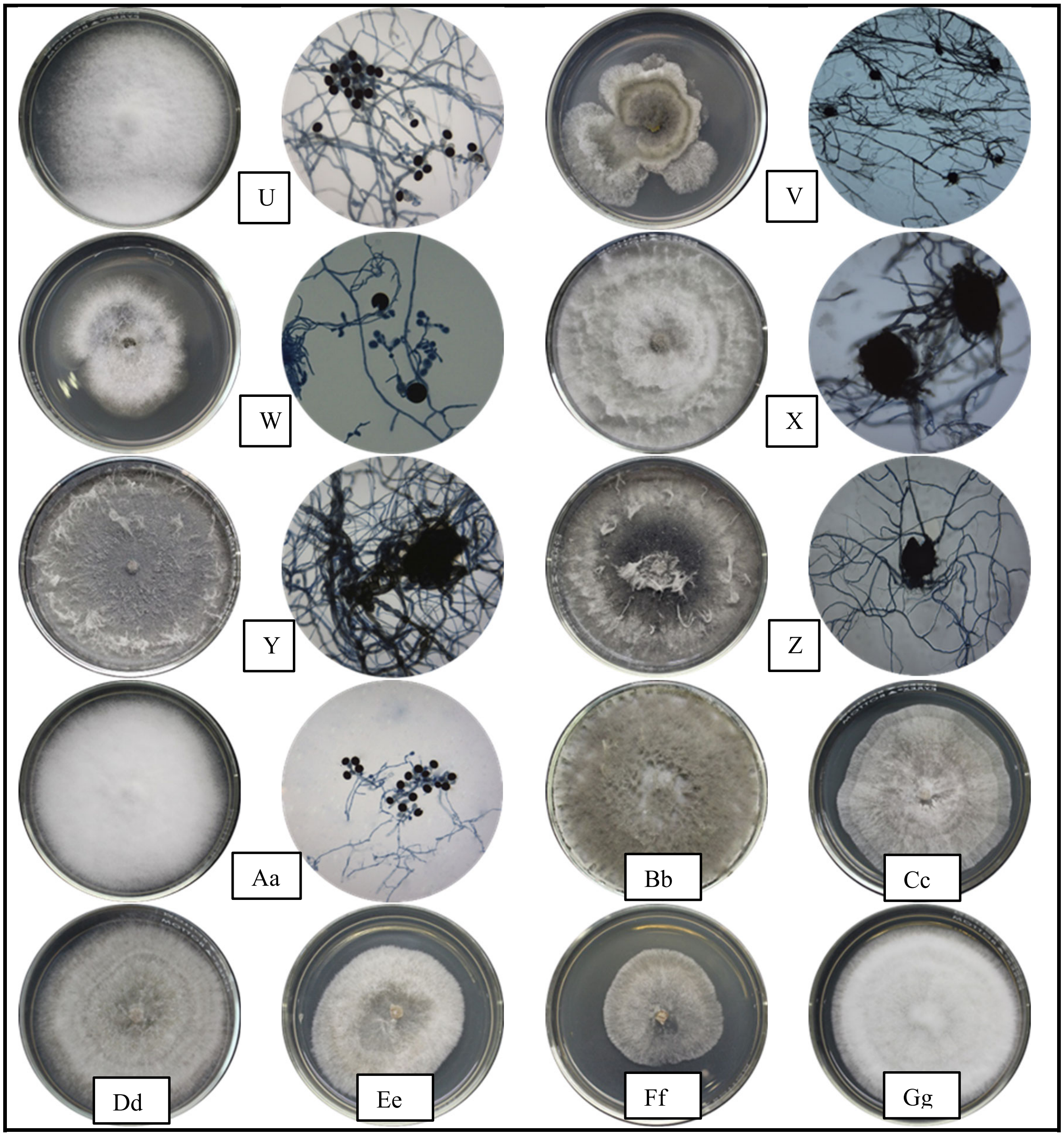

Supplementary Figure 1….continued.: U) *Nigrospora* TDC066, V) *Papulaspora*-like TDC038, W) *Nigrospora* TDC287, X) *Papulaspora*-like TDC120, Y) *Papulaspora*-like TDC137, Z) *Papulaspora*-like TDC461, Aa) *Nigrospora* TDC071, Bb) *Lasiodiplodia theobromae* TDC029, Cc) *Ceratobasidium* sp. TDC037, Dd) *Ceratobasidium* sp. TDC241, Ee) *Ceratobasidium* TDC474, Ff) NSBF TDC165, and Gg) NSBF TDC281, (400x except V at 100x).

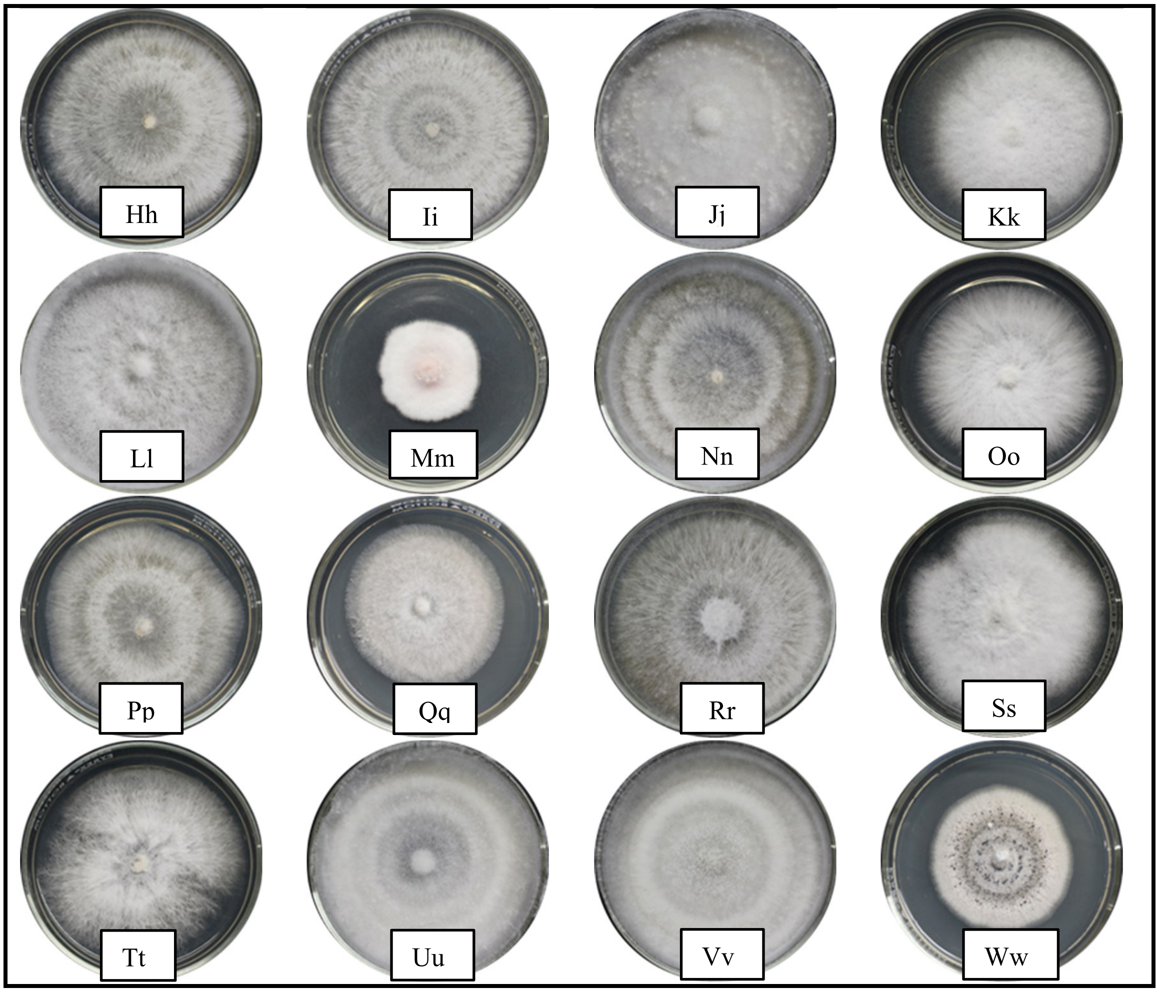

Supplementary Figure 1….continued.: Hh) NSBF TDC311, Ii) NSBF TDC292, Jj) NSBF TDC194, Kk) NSBF TDC115, Ll) NSBF TDC027, Mm) NSBF TDC691, Nn) NSBF TDC290, Oo) NSBF TDC640, Pp) NSBF TDC284, Qq) NSBF TDC195, Rr) NSBF TDC291, Ss) NSBF TDC141, Tt) NSBF TDC125, Uu) NSBF TDC264, Vv) NSBF TDC016, and Ww) NSBF TDC076.
